# A HACCP-based approach to mastitis control in dairy herds. Part 2: Implementation and evaluation

**DOI:** 10.1186/2046-0481-64-7

**Published:** 2011-03-31

**Authors:** Lies Beekhuis-Gibbon, Catherine Devitt, Paul Whyte, Luke O'Grady, Simon J More, Bairbre Redmond, Suzanne Quin, Michael L Doherty

**Affiliations:** 1 School of Agriculture, Food Science and Veterinary Medicine, University College Dublin, Belfield, Dublin 4, Ireland; 2 School of Applied Social Science, University College Dublin, Belfield, Dublin 4, Ireland

## Abstract

Part 1 of the study described the development of a Hazard Analysis and Critical Control Point (HACCP) based programme and accompanying handbook for the control of mastitis. This paper describes the implementation and evaluation of customised HACCP-based programmes, which were developed from the handbook and assessed on six Irish dairy farms. Both quantitative and qualitative (action research) research methodologies were used to measure the success of implementation and efficacy of control of sub-clinical mastitis as measured by Somatic Cell Counts (SCC) and the degree of compliance by farmers in adopting and maintaining recommendations throughout the course of the study period. No overall differences in SCC before and during the implementation of the study were found when all six farms were considered together. Three of the six study farms experienced a significant decrease in herd milk recorded SCC during the implementation of the control programme. An essential part of the study was achieving initial agreement on recommendations as well as ongoing monitoring of compliance during the study. This pilot study shows that HACCP can be implemented on farms as a means of working towards the control of mastitis and that farmer attitude, and understanding of mastitis are crucial in terms of motivation irrespective of practical approaches used to manage mastitis.

## Background

Hazard Analysis and Critical Control Points (HACCP) is now recognised as a systematic and preventive approach for identifying and controlling hazards in the food chain [1]. In recent years, its potential for application in the herd health context has also been identified [2]. One of the important indicators for milk quality is somatic cell count (**SCC**), which is mainly influenced by the incidence of clinical and subclinical mastitis. However, to date progress on preventing and controlling mastitis within dairy herds has proved difficult due to several issues including lack of knowledge transfer and proper risk-based assessment of control systems [3,4]. A HACCP-based approach may provide a useful tool for dairy farmers and their advisors.

There is growing recognition of the importance of human attitudinal factors in mastitis control and prevention [4-6]. A number of studies have examined attitudinal factors underpinning behavioural change in farmers [6,7]. A growing body of evidence-based research is helping to clarify the diverse nature of farmer motivations, and the barriers to implementation of different practices [8,9]. Kleen and Rehage [10] stressed the importance of communication skills in veterinary practice and the need to emphasise this area in the undergraduate veterinary curriculum. Consequently, a generic approach to communication when working with farmers may prove ineffective by not addressing the range of motivations and attitudes underlying behavioural change. Similarly, any such investigative approach into so called *human factors *must allow for the broad spectrum of farmers' attitudes and perceptions to emerge, particularly when acknowledging that attitudes significantly influence behavioural intentions [11]. This may explain differences in mastitis prevalence more accurately than behaviour and self-reported behaviour [7]. Effective communication between farmer and veterinarian can help address farmer attitudes and related compliance [12], mirroring similar findings in the area of human health care delivery [13,14].

A template, based on HACCP principles, for mastitis control in dairy cows was developed [15,16]. This paper (part 2 of the study) describes the implementation and evaluation of this approach. Specifically, the objectives of the present study were: (i) to customise and implement HACCP-based mastitis control programmes on six participating dairy farms following initial farm investigations; (ii) to evaluate the implementation and effectiveness of the HACCP-based programmes; and (iii) to obtain sociological insights into the human factors associated with the implementation of this programme.

## Methods

A veterinarian and social scientist conducted study field work. All participating dairy farmers (n = 6) (Table 1) were located in the east of Ireland and were selected on the basis of: (i) an acknowledgement of a SCC or clinical mastitis problem on their farm; (ii) a willingness to participate in the project; (iii) the provision of regular individual cow milk recording both during the study and for at least two years immediately prior; and (iv) farm location. Farmers were encouraged to milk record at four weekly intervals throughout the project. An initial introductory meeting addressing mastitis in general, the HACCP-based control plan and other project details, was attended by the research team and participating farmers.

**Table 1:** A summary of the general characteristics of each participating farm.

Herd ID	Breed cows (>85% cows)	Number of milking cows per year	Number of full-time positions on farm	Housing system	Mean 305 Day Milk yield (kg)	Calving pattern
1	Crossbred (50% British Friesian 50% Holstein Friesian)	68	1.3	Loose housing; Straw yard	5300	Spring 70% Autumn 30%

2	Holstein Friesian	148	2.8	Loose housing; Cubicles	7000	Spring 60% Autumn 40%

3	Holstein Friesian	150	2.5	Loose housing; Cubicles	8000	Spring 50% Autumn 50%

4	Holstein Friesian	80	1.3	Loose Housing; Cubicles	6500	Spring 60% Autumn 40%

5	Jersey	150	2.3	Loose Housing; Cubicles Straw yard (20% milking cows)	6000	Spring 20% Autumn 80%

6	Holstein Friesian	87	1.8	Loose Housing; Cubicles	7500	Spring 50% Autumn 50%

### Technical Perspectives

**Figure 1:**
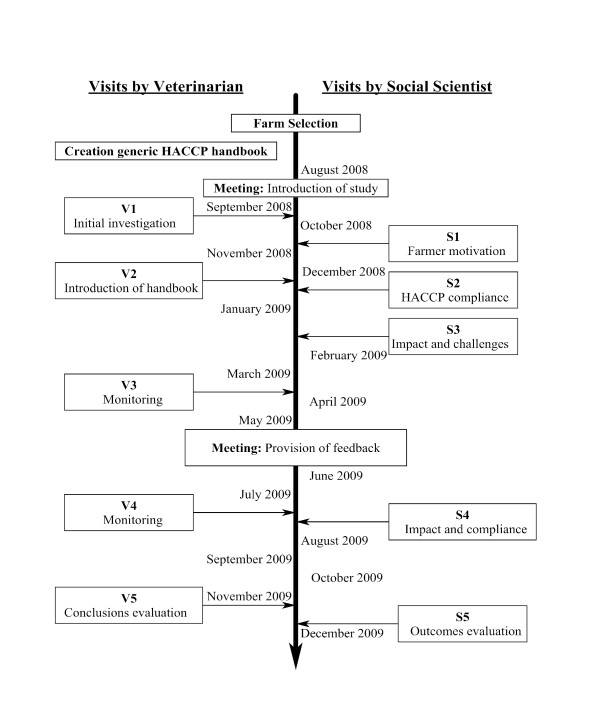
**Timeline highlighting the visits by the veterinarian and the social scientist**.

A mastitis control programme using a HACCP-based methodology based on six Critical Control Points (CCPs): udder preparation, cluster attachment, post-milking teat disinfection, milking machine monitoring, drying off, and the calving period was developed [15,16]. Each CCP consisted of control measures, monitoring points with monitoring sheets and points of verification, as well as Good Farming Practices (GFPs). Each farm was visited five times by the veterinarian over a 13-month-period (Figure 1).

#### Visit 1 [V1]

A detailed investigation was conducted at the first visit to assess farm performance of relevance to milk quality, according to methods suggested by Ruegg [17].

#### Visit 2 [V2]

A HACCP-based handbook [15,16] was customised for each farm, once the initial investigation was completed and farm-specific problems were identified. Relevant components from the HACCP-based handbook were retained with a focus on a defined set of farm-specific control measures and points of verification within each of the six CCPs. These control measures and points of verification were discussed with the farmer during the farm visit, and points of agreement or disagreement were noted.

#### Visits 3-5 [V3-V5]

On-farm compliance with the farm-specific control measures was monitored throughout the course of the project, through discussion and observation during visits 3 and 4. Further data analysis consistent with the initial investigation was also conducted. Project feedback was provided to all participants mid-project. During the final visit (V5), a range of milk quality measures were used as the basis for discussion on progress towards improved mastitis control.

### Evaluation

#### Data analysis

Changes in herd milk recorded SCC and bulk milk tank SCC were statistically evaluated per farm, calculating the differences in mean before and during implementation of the HACCP-based approach (timeline before implementation: Jan - August 2008 and timeline after onset of implementation: Jan-August 2009). For each farm, a mean and 95% confidence interval was calculated for the previous eight months and for the eight months after the onset of the programme implementation. The overall change in herd milk recorded SCC and bulk milk tank SCC was tested using the Wilcoxon matched-pairs signed-ranks test. A range of descriptive analyses was conducted to assess activity (implementation and compliance with respect to recommended control measures per CCP) on each study farm. Control measures were classified as either (i) not practiced/implemented, (ii) partially practiced/implemented, or (iii) fully practiced/implemented.

### Sociological perspectives

#### Interviews (S1-S5) and data analysis

Five interviews were conducted with participants following each farm visit as part of the action research component of the study (Figure 1). Interviews sought to investigate the farmers' views and experiences of farm visits by the veterinarian, their understanding of the HACCP-based approach, their perceptions of how it was communicated, and their reasons for compliance and non-compliance. Farmers were also invited to make recommendations for project improvement. Both the veterinarian and the social scientist met regularly throughout the study to discuss the progression of the approach, and to identify barriers to compliance on participating farms and ways in which these barriers could be addressed during visits. Interview data were analysed using three-staged thematic analysis: (i) development of basic codes that described the content of the interview, (ii) greater familiarisation with the data and subsequent development of second-level categories showing a sociological explanation of issues arising from the data, and (iii) grouping together of these categories into broad analytical themes.

## Results

### Motivations for participation

Farmer motivation to participate in the study related to a desire to reduce the incidence of clinical mastitis on their farms, reduce SCC below the level of penalisation thereby reducing the subsequent financial impact, and improve milk quality.

Initial discussions with farmers revealed differing levels of awareness of the causes of mastitis and management practices required to address these causes. Prior information and awareness, however, did not necessarily translate into practice, '*we teat spray at the moment, and we know that we're not putting on enough*' (Farmer 1). The discussions also revealed a poor understanding of the importance of subclinical mastitis and a perception that mastitis only became a '*problem*' when acute cases arose, '*I find that if I clear them up and they don't re-infect... I don't have to worry*' (Farmer 6). The impact of penalisation for high SCC served as a motivation to address mastitis; however, once SCC was below the penalisation mark, farmers felt there was less need to reduce the SCC further.

### Implementation

The initial investigations showed that chronic contagious mastitis was the primary issue on all six participating farms, and farm-specific control programmes focused on decreasing the spread of infection in the parlour, improving cure rates when possible, and preventing new infections around calving. Many farmers had difficulty implementing strategies to reduce the spread of infection in the parlour.

Only three farmers were milk recording monthly prior to the project. During the project, all farmers conducted monthly recording. As well as gauging their SCC levels, the results allowed farmers to identify high risk cows subsequently informing segregation practices, and assess levels in SCC levels - identifying outcomes/progress made while informing prevention practices and/or culling strategies, '*the milk recording has come back and it's quite good, relative to what it used to be. There're not as many high SCC cows and it may be easier to separate them now -- I'll talk to *[veterinarian] *about that...*'. More thorough investigation of these results facilitated a greater awareness of sub-clinical mastitis.

Data analysis and bacteriological investigation revealed the most important patterns of mastitis problems on each farm. This information was used to inform farmers of the necessity of carrying out control measures. Farmers reported how it informed them of the causes while identifying key areas for preventative action, specifically to their farm. These results were influential in forming the required CCPs and farmer decisions on their infrastructural set-up and milking routine, '[I found it helpful] *in relation to how contagious it is and how focused you have to be on it*'.

The use of documentation and identification of outcomes was supported by continued data analysis during the project that would show improvement, no change or a deterioration of the situation on the farm. All farmers commented favourably on the use of graphs to present feedback during visits, describing them as '*showing the results right in front of you*', '*making it more tangible*', '*accessible*' and '*specific to our farm*'.

Frequent interaction at and between visits, between farmer and veterinarian, maintained on-farm momentum and assisted in building farmer focus. However, some farmers (n = 3) reported that more frequent communication between visits would have been beneficial, '*by the 3^rd ^visit, I only felt then that I was getting into the whole thing-getting a better understanding. Then there were only two left. More visits at more regular points would have drilled home the message a bit more*.'

The HACCP-based handbook provided the template, which included control strategies based on the infectious risks of mastitis. This provided farmers with information on control measures specific to their farm. Farmers were also requested to complete monitoring sheets. Not all sections of the monitoring sheets were completed - especially in recording milk sock conditions with n = 5 farmers questioning the relevancy of completing this. Comments favoured the recording of mastitis incidence and use of teat spray as it allowed farmers to '*look at the sheet and you see it straightaway. It's focusing in on it*'... '*you can look back over time and see progress and measure things like the amount of teat spray used, and it gives an evaluation, which is important'*.

### Evaluation

**Figure 2:**
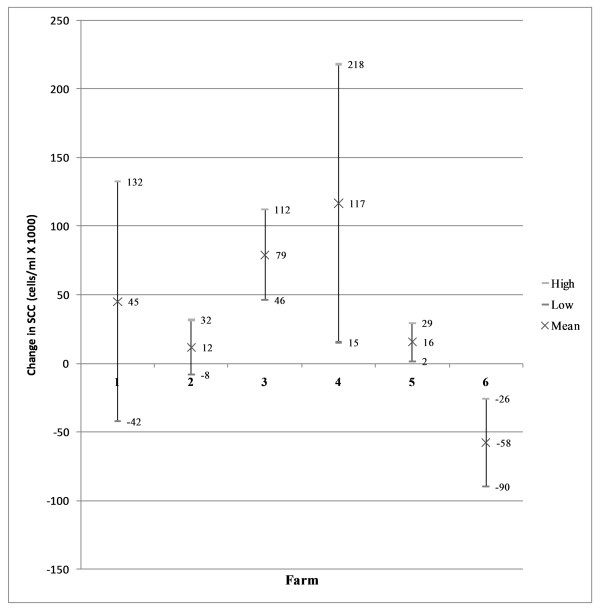
**95% confidence interval milk recorded SCC; difference in means**. The Wilcoxon Matched-Pairs Signed-Ranks Test did not detect a significant difference (p <= 0.2188) in overall change in milk recorded SCC for all farms.

**Figure 3:**
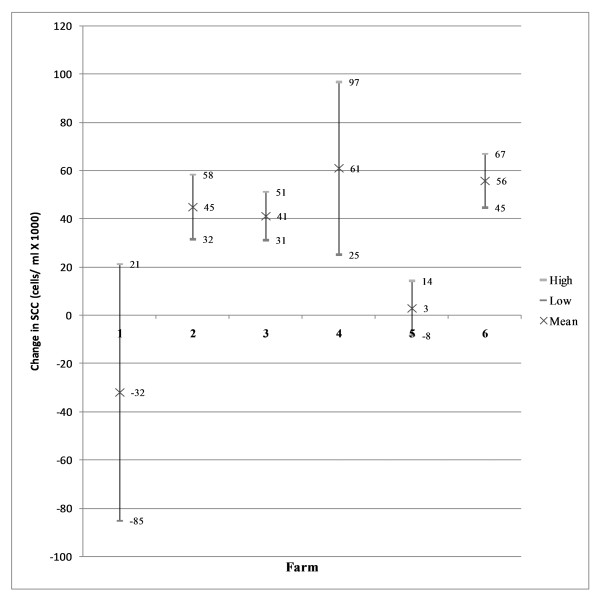
**95% confidence interval bulk milk tank SCC; difference in means**. The Wilcoxon Matched-Pairs Signed-Ranks Test did not detect a significant difference (p <= 0.09375) in overall change in bulk milk tank SCC for all farms.

The 95% confidence intervals (Figure 2) revealed that farms 3, 4 and 5 experienced a significant decrease in herd milk recorded SCC during the implementation of the control programme. Herd milk-recorded SCC did not change significantly on Farms 1 and 2. The herd milk-recorded SCC of Farm 6 increased during the implementation of the programme. The Wilcoxon Matched-Pairs Signed-Ranks Test did not detect a significant difference (p <= 0.2188) in overall change in milk recorded SCC for all farms. The 95% confidence intervals (Figure 3) of the changes in bulk milk tank SCC showed, on the contrary, that farms 2, 3, 4 and 6 had a significant decrease in SCC of the bulk milk tank and that farms 1 and 5 did not change significantly. The Wilcoxon Matched-Pairs Signed-Ranks Test did not detect a significant difference (p <= 0.09375) in overall change in bulk milk tank SCC for all farms.

There was considerable variation among farmers on the level of agreement and on subsequent adoption (given agreement) of the defined farm-specific control measures and points of verification within each of the six CCPs. Initial agreement to implement a control measure did not always translate into full implementation. Additionally, compliance rates of individual farmers were not in line with significant changes in SCC for either the milk recorded SCC or bulk milk tank SCC (Tables 2, 3, 4 and 5). Farmer 1, who was most compliant (94%, Table 2) had no significant change in SCC and Farmer 2, who was least compliant with the programme (59%, Table 2), experienced a significant reduction in bulk milk tank SCC. Farmer 6 who displayed good compliance (88%, Table 2) had a significant increase in herd recorded SCC during implementation of the programme.

**Table 2:** Number of recommendations initially agreed on and the percentage actively implemented during the study.

	Critical Control Point Number of recommendations agreed on at the start of the study for each CCP (Number of recommendations actively implemented during study)						
**Herd ID**	**Udder preparation**	**Cluster Attachment**	**Post Milking Teat Disinfection**	**Functioning Milking Machine**	**Drying off process**	**Calving**	**Total number agreed on per farm (% implemented)**

1	4 (4.0)	9 (8.5)	3 (3.0)	2 (1.0)	3 (3.0)	4 (4.0)	25 (94)

2	2 (0.5) ^a^	9 (4.5)	3 (1.5)	2 (1.5)	3 (3.0)	4 (2.5)	23 (59)

3	0 (0.5)	7 (5.0)	3 (2.5)	2 (1.5)	3 (3.0)	3 (2.0)	18 (81)

4	2 (2.5)	9 (6.5)	3 (2.5)	2 (1.0)	3 (3.0)	4 (4.0)	23 (85)

5	3 (2.5)	9 (6.5)	3 (2.0)	2 (2.0)	3 (3.0)	4 (3.5)	24 (81)

6	2 (2.0)	8.5 ^a ^(6.5)	3 (3.0)	2 (1.5)	3 (3.0)	3 (3.0)	21.5 (88)

Average compliance rate per farm per CCP (%)	68	69	81	71	100	86	

^a ^The decimal .5 indicates partial agreement or compliance with recommendations

**Table 3:** Practicing and recommended control measures for each Critical Control Point (CCP) 1 and 2 at Visit 1, Visit 2 and post Visit 2.

	Visit 1^a^			Visit 2^b^				Post Visit 2^c^		
	
	NP	PP	P	NR	RNA	RPA	RA	NI	PI	I
*CCP1. Udder preparation*										
Washing	4	2	0	6	0	0	0	6	0	0
Drying	4	2	0	6	0	0	0	6	0	0
Foremilking	3	1	2	0	1	0	5	1	0	5
Predipping	5	0	1	3	2	0	1	3	2	1
Drying with paper towel, towel per cow	4	0	2	0	4	0	2	2	3	1
Monitoring sheet milksock	6	0	0	0	1	0	5	2	2	2
										
*CCP2. Cluster attachment*										
Adequate plant hygiene	0	2	4	0	0	0	6	0	4	2
Monitoring sheet with washing protocol milking machine	5	0	1	0	0	0	6	2	2	2
Quality of rubberware	2	0	4	0	0	0	6	1	0	5
Monitoring sheet frequency changing liners	6	0	0	0	0	0	6	2	1	3
Segregation/cluster dipping	4	2	0	1	0	1	4	1	4	1
Monitoring sheet segregation	6	0	0	1	0	0	5	5	0	1
Correct method of cluster attachment	0	0	6	0	0	0	6	0	0	6
Correct balancing of clusters	0	1	5	0	0	0	6	0	0	6
Correct method of cluster removal	0	0	6	0	0	0	6	0	0	6

^a ^NP = Not practicing, PP = Partially practicing, P = Practicing

^b ^NR = Not recommended, RNA = Recommended but not agreed, RPA = Recommended and partially agreed, RA = Recommended and agreed

^c ^NI = Not implemented, PI = Partially implemented, I = Implemented

**Table 4:** Practicing and recommended control measures for each Critical Control Point (CCP) 3, 4, 5 and 6 at Visit 1, Visit 2 and post Visit 2.

	Visit 1^a^			Visit 2^b^				Post Visit 2^c^		
	
	NP	PP	P	NR	RNA	RPA	RA	NI	PI	I
*CCP 3. Post milking teat disinfection*										
Carrying out teat dipping/teat spraying	0	0	6	0	0	0	6	0	0	6
Good quality teat spraying/dipping	2	1	4	0	0	0	6	0	2	4
Monitoring sheet quantity and brand teat disinfectant used	6	0	0	0	0	0	6	2	1	3
										
*CCP 4. Milking machine functioning*										
Adequate functioning milking machine	0	3	3	0	0	0	6	0	2	4
Monitoring sheet: milking machine report	6	0	0	0	0	0	6	2	2	2
										
*CCP 5. Drying off process*										
Drying off protocol	1	0	5	0	0	0	6	0	0	6
Teat preparation	0	0	6	0	0	0	6	0	0	6
Monitoring sheet treatment protocol	5	0	1	0	0	0	6	0	0	6
										
*CCP 6. The calving period*										
Calving shed hygiene	0	5	1	0	0	1	5	0	2	4
Calving shed lay out	2	3	1	0	0	1	5	1	3	2
Calving shed stocking density	1	3	2	1	0	0	5	1	1	4
Calving hygiene	0	0	6	0	0	0	6	0	0	6

^a ^NP = Not practicing, PP = Partially practicing, P = Practicing

^b ^NR = Not recommended, RNA = Recommended but not agreed, RPA = Recommended and partially agreed, RA = Recommended and agreed

^c ^NI = Not implemented, PI = Partially implemented, I = Implemented

**Table 5:** Practicing and recommended Good Farming Practices (GFPs) at Visit 1, Visit 2 and post Visit 2.

	Visit 1^a^			Visit 2^b^				Post Visit 2^c^		
	
	NP	PP	P	NR	RNA	RPA	RA	NI	PI	I
Clean teats when entering the parlour	1	2	4	0	0	0	6	0	3	3
Clean lying areas and walkways	0	4	2	0	0	0	6	0	2	4
Prevent mud pooling in field	0	1	5	0	0	0	6	0	0	6
Availability and use of disposable paper towels in parlour	4	0	2	0	0	0	6	3	2	1
Wear gloves during milking	0	2	4	0	0	0	6	0	2	4
Let cows stand 30 minutes after milking	2	1	3	0	0	0	6	0	3	3
Abrupt drying off	1	1	4	0	0	0	6	0	1	5

^a ^NP = Not practicing, PP = Partially practicing, P = Practicing

^b ^NR = Not recommended, RNA = Recommended but not agreed, RPA = Recommended and partially agreed, RA = Recommended and agreed

^c ^NI = Not implemented, PI = Partially implemented, I = Implemented

### Assessments of implementation

On each farm, the defined control measures included a combination of GFPs and mastitis-specific strategies. As highlighted in Tables 3, 4 and 5, the GFPs were less likely to be implemented than the mastitis-specific strategies if they had not already been carried out before the start of the study.

**Figure 4:**
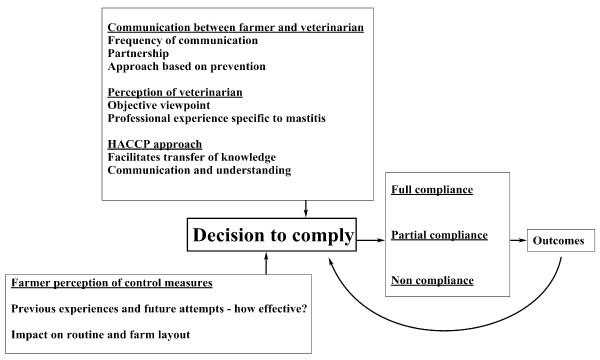
**Sociological factors taken into account during compliance with implementation of the HACCP-based control measures**.

Despite varying levels of compliance, all farmers when deciding whether or not to comply with the recommendations took similar factors into account. These decisions were influenced by whether or not similar control measures had been implemented on the farm previously (Figure 4), and how effective these were in addressing the mastitis problem, '*I tried dipping before but didn't really see any results, I wasn't getting penalised either so I guess I wondered what was the point*'. The positive and negative impacts that such actions would have on the farm routine and the feasibility of alternatives were also important in determining compliance, '*segregating is not an option for us - it takes too long in the parlour, the best thing to do is to identify the top ten high-cell-count cows and disinfect their clusters so it isn't being transferred over to other animals, which we're starting to do now*'. The availability of on-farm resources in line with the routine and the infrastructural set-up on the farm and how this would facilitate implementation of a control measure was deemed important, '*well at the moment it is not feasible to leave cows standing for 30 minutes in the yard, as I've groups coming in and out-it just wouldn't work*'. Further the position of the farmer in the context of milk quality penalties was important, '*I thought it was time consuming, I didn't feel that it was necessary. Our results aren't too bad at the minute, so why add an extra load of work when you don't need to*'. Drawing on previous experiences of implementing controls and the observed outcomes prior to the study, farmers also considered the possible efficacy of control measures for addressing the mastitis problem and whether or not this would justify the required financial investments and changes in routine required, '*and one of the recommendations was to dip... and then you go through a year and there's no progress'*. Alternatives to control measures that farmers reported as not possible to implement were developed in partnership with the farmer. These alternative measures, e.g. segregation of 10 high SCC cows, were oriented towards reducing the mastitis problem. Though this proved beneficial, it meant that in some cases, over-emphasis was placed on control measures that were feasible to the detriment of those that farmers decided were not possible to implement, e.g. segregation of all problem animals.

The identification of outcomes and results from the implementation of control measures was important in further motivating the continued implementation of control measures. *'We have improved on last year, when there were six or seven *[incidents of clinical mastitis]*. So it would be interesting to look at those records. It would motivate me and the staff, to say, okay, we've had two heifers with mastitis and last year there were 6 or 8. So it would keep us focused on doing the job right. We certainly want to get our cell count below 200, whereas previously we would have been satisfied with 300.' ... 'The milk recording has come back and it's quite good... there're not as many high cows... we have been dipping the clusters of those cows and I might maintain that.'*

## Discussion

This paper investigated the implementation and evaluation of the efficacy of a HACCP-based approach for the control of mastitis on six Irish dairy farms from a technical and sociological perspective. Overall, the programme was useable and verifiable on all participating farms. However, the control of mastitis on the participating farms generally reflected the variable compliance among farmers.

The initial investigative visit was essential as it allowed the veterinarian to identify specific problems on each farm so that relevant recommendations could be made. There were varying levels of compliance throughout the course of the study, reflective of the willingness of the farmers to translate their motivations into practice. For example, Farmer 2 was very reluctant to implement most of the recommended control strategies from the initial commencement of the study. On the other hand, Farmer 1 realised from the outset that preventative measures were fundamental to improving SCC in the long term.

The lack of a consistent relationship between compliance rates and significant changes in SCC could be attributed to various factors. Farmer 1, who had the highest compliance rate, did not experience any significant changes in SCC of the bulk milk tank or herd milk recording. This could be contributed to a change of calving seasons (from all spring calving to spring and autumn calving) in the middle of the project and the change of milk processor (the new milk processor was a lot less vigilant in hygienic standards and feedback of information). Farmer 2, whose compliance rate was the lowest over all participants, still managed to decrease the bulk milk tank SCC significantly which indicates that even partial compliance with the programme resulted in improved management of the bulk milk tank. This was certainly the case for Farmer 6, who had a significant increase in herd milk recorded SCC and a significant decrease in SCC of the bulk milk tank. However his selective adoption of focusing on the segregation of 10 high SCC problem cows, was helping him to score high on compliance, even though it was only a short-term strategy.

An increasing number of studies recognise the impact of human attitudinal factors on mastitis prevention measures [6,7,18]. The inclusion of a social science approach in the study facilitated the identification of farmer motivation and attitudes, and the related factors that influenced decisions to implement control measures. Farmer perceptions and experiences of the communication between veterinarian and farmer and the facilitating mechanisms of the HACCP-based approach influenced their decisions to comply with study recommendations (Figure 4). A study of an Australian national mastitis control programme, "Countdown DownUnder", by Brightling [19], provides evidence pointing towards the importance of the communication process in helping farmers understand the issues at hand - in order to improve compliance, particularly in the communication of information that is often equivocal. Regular communication (through visits and phone calls) contributes to a greater understanding of the objectives of the study and provides the farmer with the opportunity to raise issues of concern or seek clarification, resulting in greater compliance.

The transfer and communication of knowledge is significant in influencing compliance. The information and recommendations communicated were validated by farmers' perceptions of the veterinarian's level of expertise specific to mastitis. The relationship between veterinarian and farmer in agreeing targets was also important, '*I had input as well into the targets we set, which was important. Because you have to take into account the farm and your own situation. So it's important that the farmer has input into the targets*'. Farmers reported throughout the project that the HACCP-based approach allowed them to develop a greater understanding of mastitis as a disease and of its specific situation to their individual farm; this approach increased focus and awareness, and encouraged a sense of greater vigilance on a daily basis.

Regular communication and the opportunity for negotiation between veterinarian and farmer in this study were facilitated by the relatively frequent visits and contact. It would have been beneficial to visit the farms more frequently e.g. once a fortnight but for practical reasons, unfortunately this was not possible. However, the approach adopted was beneficial in maintaining focus and in the development of greater awareness on the necessity of daily vigilance. This change in attitude among the participating farmers during the study was largely facilitated by the relationships formed between farmers and the veterinarian. Although this finding was beyond the scope of this study, it does emphasise the importance of well-founded relationships and ongoing communication in the herd health sector. The process was also facilitated by the customised HACCP-based handbook, which provided farmers with information specific to their farm and allowed them to agree on and note what control measures would be implemented. However, despite this, compliance with all control measures was not achieved on all participating farms.

In the present study, control measures were fully implemented, partially implemented or not implemented at all. Partial implementation was judged on the basis of the frequency and quality of implementation of the control measure. Greater compliance with suggested control strategies may have been achievable with a restructuring of the approach adopted during the study through more frequent farm visits and on-farm observations that would allow ideal coaching by the veterinarian and a greater transfer and intensity of information sharing. Farmers spoke of how frequent visits and communication provided a channel for the development of focus and awareness on their mastitis problem, with some farmers being compelled to complete certain measures because of upcoming visits. Repeating this study with a large group of farms would strengthen the value of performed statistics and could help detecting changes in SCC more successfully.

Sociological insights revealed the barriers identified by farmers when considering compliance with a control measure. These barriers were discussed with the farmer and more feasible, alternative approaches were identified and implemented, resulting in greater compliance. Leeuwis [20] argues that effective innovation involves developing a social learning process that includes negotiation, allowing all involved to reach a shared common view on desired goals, responsibilities and standards. The flexibility of this study approach was beneficial as it did allow farmers to negotiate and adjust certain control measures to adopt into their farm routine and infrastructural challenges whilst not reducing the overall planned effectiveness of the control programme. Although the HACCP-based approach allowed a degree of flexibility in the implementation of control measures, alternative measures should not be viewed as quick solutions that distract the farmer from key control measures.

Farmers were less likely to implement longer-term control strategies; for example, methods to decrease in-parlour spread of infection. This reflects farmers' reliance on the expectation of short-term positive results to justify the implementation of certain practices. This was certainly observed in the changes of SCC, which were overall not significant. This study showed that positive results, such as a reduction in the incidence of clinical mastitis or SCC played a significant role in informing farmers' decisions to continue with the implementation of a control measure. Notably, this study took place over a one-year period, arguably too short a time for considerable changes, if any, on the six farms. A discontinuation of the HACCP-based practices during the study, were related to the lack of beneficial outcomes and observable results on the farm. In light of this, therefore, it is necessary in a study of this nature to consider how best to manage farmer expectations in terms of their reliance on observable outcomes, noting the time period in which any such results could be experienced. The study farms were broadly representative of commercial Irish dairy farms within which herd health and production management programmes are generally not well developed. The recent creation of a national dairy herd health initiative (Animal Health Ireland) will radically change this situation in the future [21]. However, there is little doubt that risk management programmes such as that developed in this study would be easier implemented on farms with established herd health and production management programmes [2].

The inclusion of information on specific topics such as subclinical mastitis in the customised HACCP- based handbook may have provided greater awareness and transfer of information on these issues. Overall, a lack of awareness on the prevalence of subclinical mastitis was apparent throughout - this was augmented by the failure of some farmers to milk record on a frequent basis. Increased discussion on the economic impact of high SCC would have encouraged farmers to move away from reliance on the point of penalisation (400,000 cells/ml) as a benchmark for somatic cell count levels. The customised HACCP-based handbook provided a means for information and monitoring of control measure-related activities. The failure to complete all monitoring sheets and the prevalence of transferring of data from other sources among some farmers may point to the need for readjustment of, and greater communication on, the purposes of this handbook [14], while an increase in coaching frequency could have improved the completion rate of the monitoring sheets. While they were supportive of the project and kept informed of the study, the research was conducted without the active participation of the farmers' private veterinary practitioners. Active involvement of the practitioners, after a period of initial training, may have had a beneficial effect by allowing increased frequency of coaching and reinforcement of the HACCP-based approach.

## Conclusion

This pilot study shows that HACCP can be implemented on farms as a tool to facilitate the control of mastitis. The associated structured approach with monitoring, implementation, and verification points is useful in formalising controls while still being flexible enough to enable it to be tailored for specific farms. This flexibility is achieved by the customisation of the HACCP-based handbook relevant to each farm situation. The overall study approach (including visit frequency, farmer communication, farmer coaching, and action research) encouraged a greater understanding of the issues at hand.

## Conflict of interests statement

None of the authors has any financial or personal relationships that could inappropriately influence or bias the content of the paper.

## Authors' contributions

LB-G collected and analysed the farm-level mastitis data and was the lead person in the field research. CD collected and analysed the farm-level sociological data. PW provided specific expertise in relation to HACCP principles. LOG, SM and MD provided expertise on bovine health management and epidemiology as it applies to mastitis control. BR and SQ provided expertise in the area of participatory research. All authors read and approved the final manuscript.
